# Supplementary material for: Microbe-ID: an open source toolbox for microbial genotyping and species identification
Source: PeerJ. 2016 Aug 18;4:e2279. doi: 10.7717/peerj.2279 (PMC4994078; doi:10.7717/peerj.2279)
Supplement: Figure S1 — The interface includes tabs for input of barcodes and a dropdown help link that contains laboratory protocols, examples data, and logs of each query dataset. The input frame has a control scheme that only permits data in FASTA format. [file peerj-04-2279-s001.pdf]

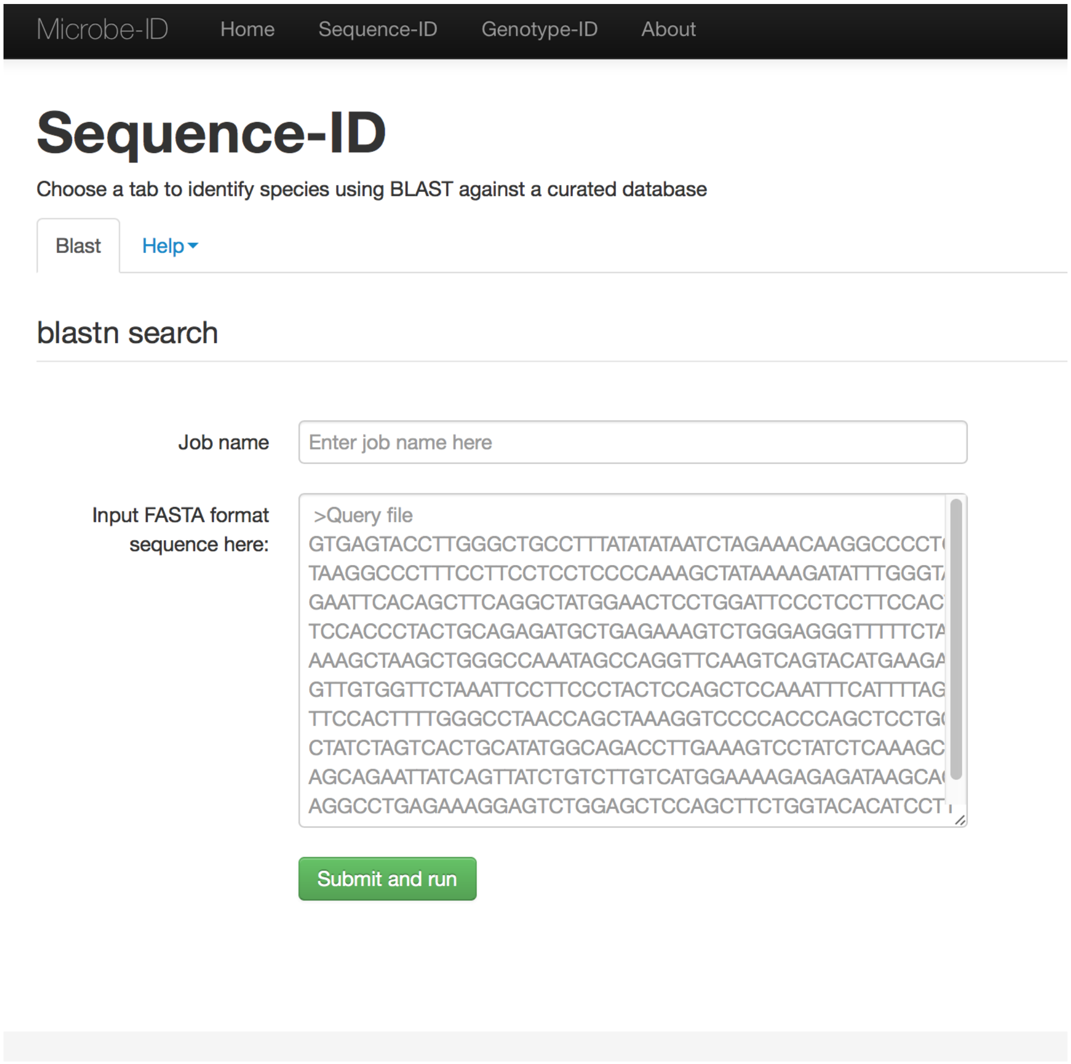

Supplementary Figure 1. Screen capture of the Sequence-ID user interface of Microbe-ID. The interface includes tabs for input of barcodes and a dropdown help link that contains laboratory protocols, examples data, and logs of each query dataset. The input frame has a control scheme that only permits data in FASTA format.
