# Supplementary material for: Microbe-ID: an open source toolbox for microbial genotyping and species identification
Source: PeerJ. 2016 Aug 18;4:e2279. doi: 10.7717/peerj.2279 (PMC4994078; doi:10.7717/peerj.2279)
Supplement: Figure S2 — Shown is the SSR-ID module for P. ramorum. Genotype-ID includes an Instructions link with an example Excel file that the user can modify, copy, and paste into the data input form. There is no limit to the number of queries submitted. In the Analysis section, tabs are provided to select either between a distance tree or a minimum spanning network rendering. [file peerj-04-2279-s002.pdf]

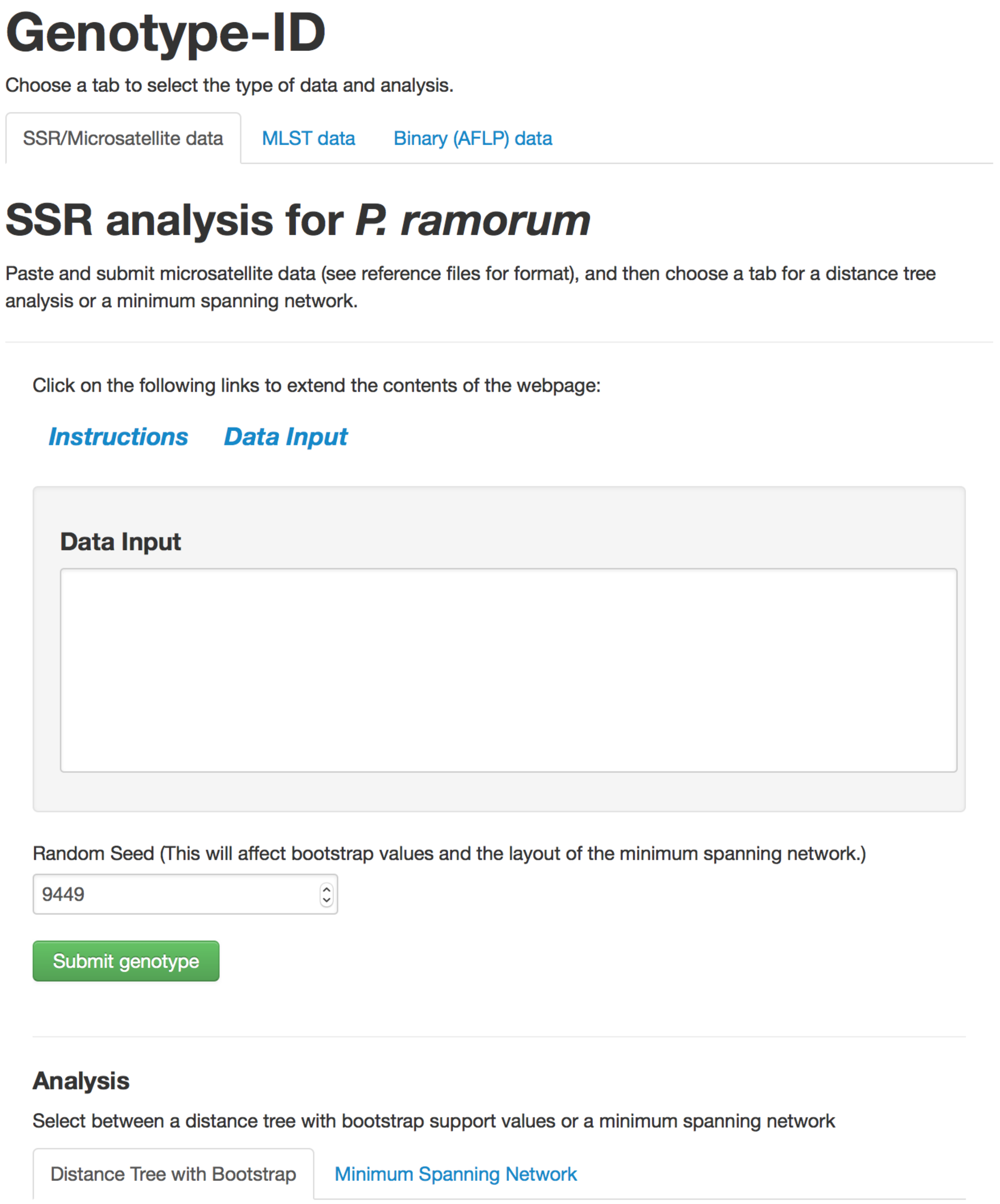

Supplementary Figure 2. Screen capture of the Genotype-ID user interface of Microbe-ID. Shown is the SSR-ID module for P. ramorum. Genotype-ID includes an Intstructions link with an example Excel file that the user can modify, copy, and paste into the data input form. There is no limit to the number of queries submitted. In the Analysis section, tabs are provided to select either between a distance tree or a minimum spanning network rendering.
