# Supplementary material for: Microbe-ID: an open source toolbox for microbial genotyping and species identification
Source: PeerJ. 2016 Aug 18;4:e2279. doi: 10.7717/peerj.2279 (PMC4994078; doi:10.7717/peerj.2279)
Supplement: Figure S4 — The queries used for this iteration of Binary-ID are two samples from a presumptive “Athena” origin. Queries are represented in red. Note that all queries were correctly placed amongst samples of its presumptive clonal lineage while also representing relationships between lineages in the reference dataset, indicating both queries are more closely related to the “Athena” population than the “Mt. Vernon” population. [file peerj-04-2279-s004.pdf]

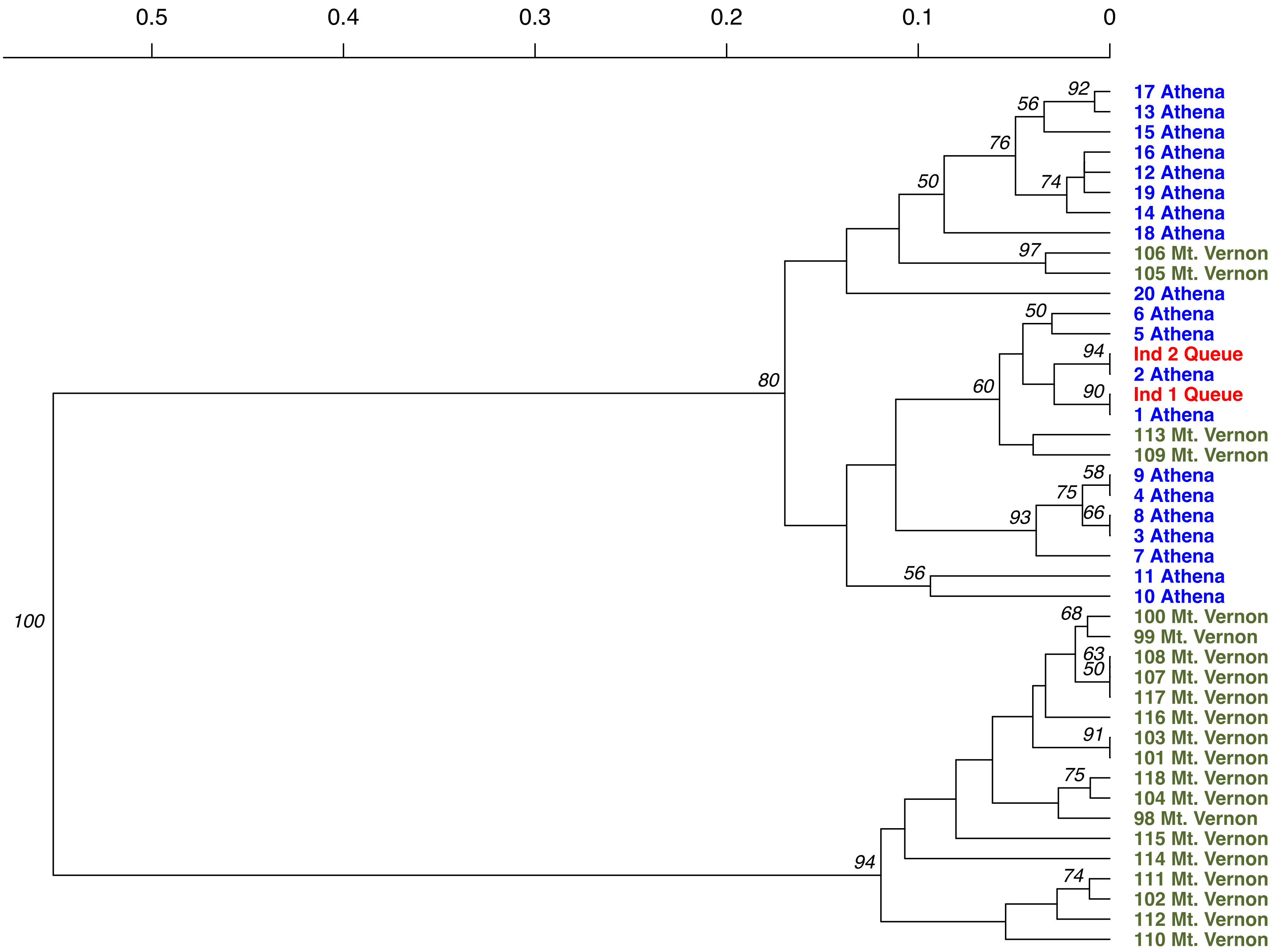

Supplementary Figure 4. UPGMA dendrogram of sub-module Binary-ID of Genotype-ID using 100 bootstrap replicates for *Aphanomyces euteiches.* The queries used for this iteration of Binary-ID are two samples from a presumptive “Athena” origin. Queries are represented in red. Note that all queries were correctly placed amongst samples of its presumptive clonal lineage while also representing relationships between lineages in the reference dataset, indicating both queries are more closely related to the “Athena” population than the “Mt. Vernon” population.
