# Supplementary material for: Microbe-ID: an open source toolbox for microbial genotyping and species identification
Source: PeerJ. 2016 Aug 18;4:e2279. doi: 10.7717/peerj.2279 (PMC4994078; doi:10.7717/peerj.2279)
Supplement: Figure S3 — The tree was constructed using 100 bootstrap replicates and MLST analysis of 8 genes (5 housekeeping genes and 3 virulence genes) of Clavibacter michiganensis. The query used is a concatenation of a WASH sample using all 8 genes. Queries are represented in blue. Note that all queries are correctly placed amongst samples of its presumptive clonal lineage while also representing relationships between lineages in the reference dataset. [file peerj-04-2279-s003.pdf]

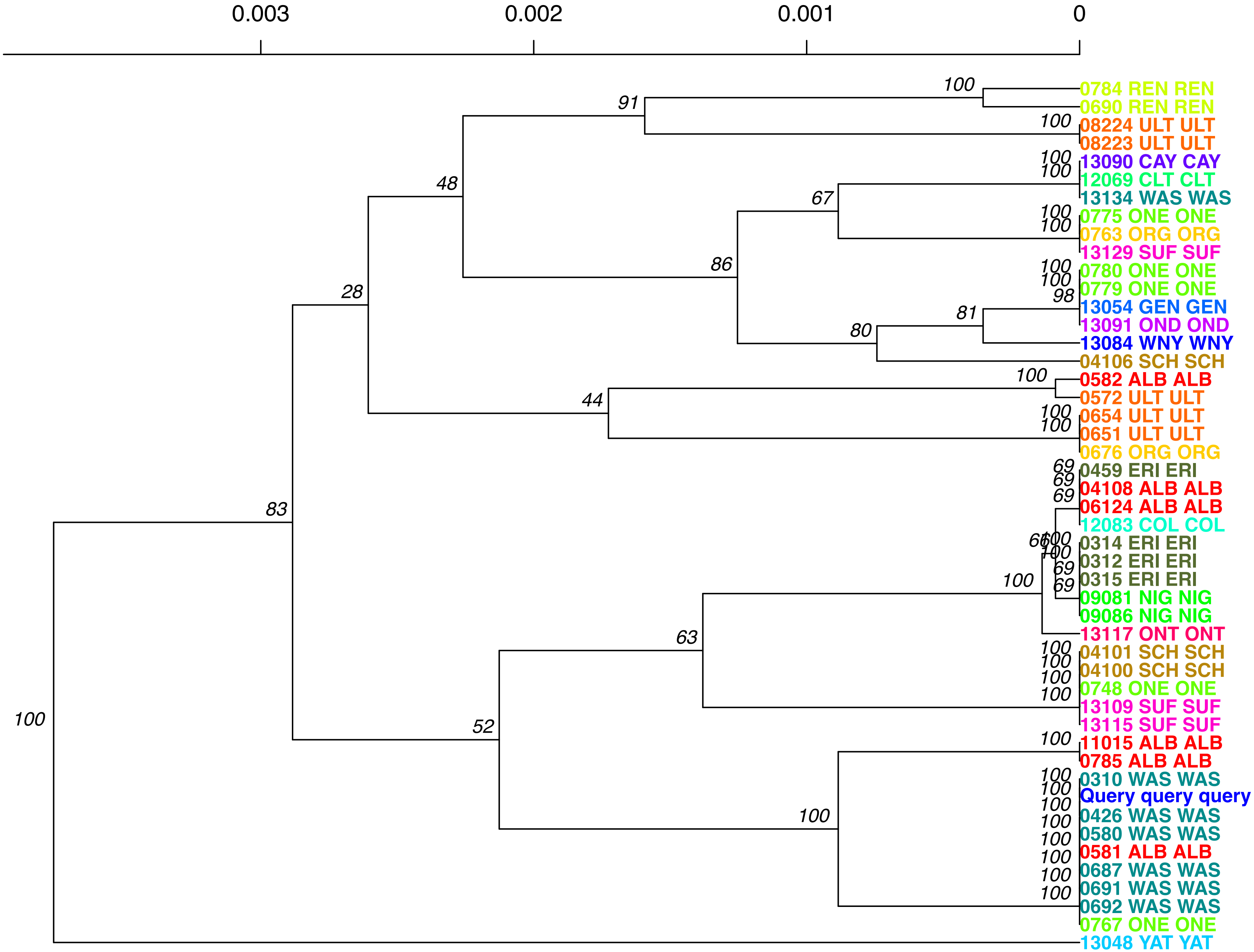

Supplementary Figure 3. UPGMA dendrogram of sub-module MLST-ID of Genotype-ID. The tree was constructed using 100 bootstrap replicates and MLST analysis of 8 genes (5 housekeeping genes and 3 virulence genes) of *Clavibacter michiganensis*. The query used is a concatenation of a WASH sample using all 8 genes. Queries are represented in blue. Note that all queries are correctly placed amongst samples of its presumptive clonal lineage while also representing relationships between lineages in the reference dataset.
